# Boundary representation of neural architecture and connectivity

**DOI:** 10.1186/1471-2202-12-S1-P59

**Published:** 2011-07-18

**Authors:** Mario Negrello, Ivan Raikov, Erik De Schutter

**Affiliations:** 1 Computational Neuroscience Unit, Okinawa Institute of Science and Technology, Okinawa, Japan; 2 University of Antwerp, Antwerp, Belgium

## 

The general objective of this work is to develop a description language for constructive 3D boundary representation [5] of neuroanatomical structures and connectivity at various levels of granularity (from coarse-resolution solids to fine meshes).

This approach is motivated by a desire to capture regularities in neural circuitry as revealed by neuro-architectonic studies [4], while at the same time to explore hypotheses about anatomic variability of various origins, such as experimental uncertainties, cross species scaling factors, individual differences, and assess them for their impact on connectivity, and ultimately on network dynamics.

Boundary representation is a general approach to describe 3D objects solely by their surface. Boundary representations consist of topological objects and their concrete geometrical representations in terms of enclosing boundaries. Topological elements include vertices, edges and faces, with corresponding geometrical elements being points, curves, and surfaces. The relationships between topological elements in a structure are expressed by means of a graph of topological connections.

The description language is being implemented on top of the GNU Triangulated Surface library [3], and provides the ability to:

• Specify compound topological objects with parametric geometry;

• Specify geometric parameters for the instantiation of topological objects, such as coordinates for placement, or probability distributions for random placement of a group of identical objects;

• Specify individual coordinate systems for different cell populations;

• Define categories of topological objects, such as stellate, basket and Golgi cells, which may be part of a morphological continuum;

• Define rules for connectivity between different categories of objects.

We present the anisotropic cerebellar circuitry as a case study, and define boundary representations of the arrangement of Purkinje and Golgi cells in the cerebellar cortex. We use the hexagonal grid pattern suggested by Palkovits et al. in [1,2], while allowing for small variability in the placement of cells within a hexagon.

The implementation of the language instantiates the topological objects, computes the intersections of the resulting surfaces, and given connectivity rules for the different categories of objects, computes the potential synaptic connectivity (producing graph theoretical measures, as well as connectivity histograms), and ultimately aims to generate connectivity descriptions for the NEURON simulator.
